# From NHANES 2005–2017: Weight-adjusted waist index and diabetic retinopathy among the general U.S. population

**DOI:** 10.1097/MD.0000000000045110

**Published:** 2025-10-10

**Authors:** Junxian Lei, Kun Lian, Pai Zhou, Jun Peng, Qinghua Peng

**Affiliations:** a Hunan University of Chinese Medicine, Changsha, Hunan, China; b Hunan Engineering Technology Research Center for the Prevention and Treatment of Otorhinolaryngologic Diseases and Protection of Visual Function with Chinese Medicine, Changsha, Hunan, China.

**Keywords:** cross-sectional study, diabetes, diabetic retinopathy, NHANES, weight-adjusted waist index

## Abstract

This research sought to investigate the link between the weight-adjusted waist index and diabetic retinopathy (DR). Data from the National Health and Nutrition Examination Survey spanning 2005 to 2018 were utilized in this cross-sectional study. Using multivariable logistic regression, the relationship between the weight-adjusted waist index and DR was examined. Moreover, generalized additive models as well as smooth curve fitting techniques were used. This cross-sectional study involved 2001 individuals, comprising 1031 men and 970 women, with an average age of 59.4 ± 13.8 years. The comprehensive model, accounting for multiple variables, indicated an inverse relationship between the weight-adjusted waist index and DR (OR = 1.32, 95% CI: 1.02–1.70). Subgroup analyses revealed a positive link between the weight-adjusted waist index and DR among women, individuals aged 50 and above, and Non-Hispanic Whites. Furthermore, we identified both the direct and inverse relationships between the weight-adjusted waist index and DR by creating a smooth graph. In the general American population, the weight-adjusted waist index is positively associated with DR according to our research. Future studies should investigate the impact of the weight-adjusted waist index on diabetic complications and identify possible mechanisms.

## 1. Introduction

Diabetic retinopathy (DR) represents a significant complication associated with diabetes mellitus, with a global prevalence estimated at 35% among individuals with diabetes.^[1]^ As diabetes becomes more common, DR continues to be a leading cause of vision loss in many industrialized countries.^[2]^ Up to 160.5 million individuals worldwide are likely to suffer from DR, according to The Vision Loss Expert Group.^[3]^ The clinical significance lies in preventing and treating DR in a timely manner.

Obesity constitutes a significant global public health threat and is intricately associated with numerous prevalent diseases, including diabetes and its related complications.^[4]^ Historically, body mass index (BMI) has been used to measure obesity, but it fails to distinguish between muscle and fat.^[5]^ Waist circumference (WC) has been established as a reliable predictor of obesity, demonstrating a significant association with visceral fat and abdominal adiposity.^[6]^ Nonetheless, research has shown a strong link between WC and BMI, which reduces the effectiveness of WC as a standalone indicator of obesity.^[7]^ Visceral fat has recently been identified as a more accurate marker of adverse metabolic profiles, especially those associated with abdominal obesity.^[8]^ Accordingly, Park et al developed a new adiposity metric, the weight-adjusted waist index.^[9]^ By using the weight-adjusted waist index, weight-independent centripetal obesity can be elucidated, which reduces its correlation with BMI and thereby emphasizes the advantages of WC.^[10]^ Recent research has shown a link between the weight-adjusted waist index and the rates of diabetes, along with overall and cardiovascular death rates.^[11,12]^

DR is associated with obesity as a major risk factor,^[13]^ and controlling weight through physical activity, dietary management, and other measures is acknowledged for its substantial preventive and therapeutic benefits.^[14]^ Nonetheless, the link between the weight-adjusted waist index and the DR remains largely unexplored.

As a result, the purpose of this study was to examine the relationship between weight-adjusted waist index and DR among adult Americans using data from the National Health and Nutrition Examination Survey (NHANES).

## 2. Materials and methods

### 2.1. Study population

**Figure 1.**
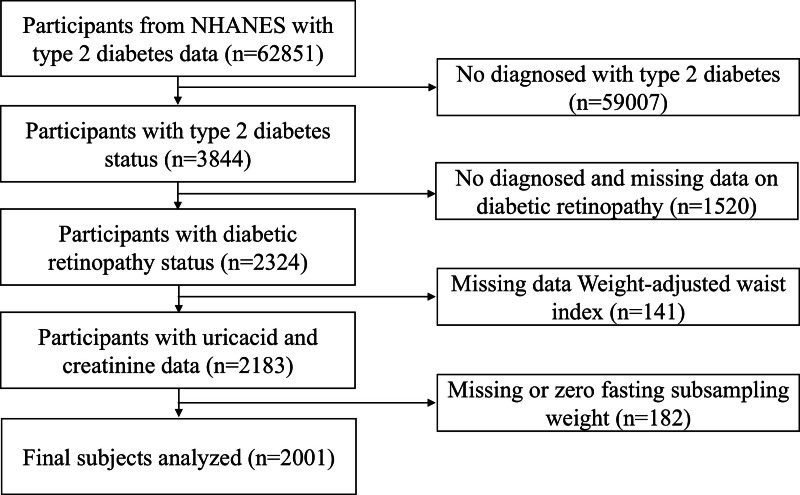
Flowchart of the participant selection. NHANES = National Health and Nutrition Examination Survey.

Using a stratified sampling technique, NHANES gathers extensive information from a sample that reflects the nation’s population. This approach aims to furnish objective and accurate statistics pertaining to the health status of the population and to address emerging public health concerns. In the present study, data from the 2005–2018 NHANES encompassing 70,190 participants were analyzed. Exclusions were made for 7339 individuals with missing diabetes or fasting blood glucose data, 59,007 individuals without a diabetes diagnosis, 1520 individuals lacking or with missing DR data, 141 individuals with incomplete diabetes data on the weight-adjusted waist index, and 182 individuals with missing data or 0 fasting subsample weight. Consequently, the final analytical sample comprised 2001 participants (Fig. 1).

### 2.2. Evaluation of diabetes mellitus and vision problems in diabetics

Diabetes was identified using these standards: pre-diagnosis by a medical expert, a fasting blood sugar level of more than 7.0 mmol/L, HbA1c levels above 6.5%, or the use of diabetes medication.^[15]^ A binary self-reported question confirmed diabetic eye damage, showing that a physician had told the participant their vision was impacted by diabetes.^[16]^

### 2.3. Weight-adjusted waist index measurement

With the weight-adjusted waist index, both WC and body weight are combined to assess obesity. When weight-adjusted waist index scores are elevated, obesity is more prevalent. At the Mobile Examination Center, trained health professionals measured WC and body weight. We used the square root of each individual’s weight in kilograms divided by the circumference of their waist in centimeters for our analysis of the weight-adjusted waist index.

### 2.4. Covariates

This research took into account demographic factors such as age, ethnicity, sex, BMI, level of education, and income-to-poverty ratio. The research included various anthropometric and lab variables, such as fasting glucose, HbA1c, albumin, triglycerides, total cholesterol, HDL, and LDL. Individuals provided information about their everyday routines and health status, including activities like smoking and drinking, along with physiological metrics such as blood pressure. Low cotinine concentrations (<0.015 ng/mL), moderate cotinine concentrations (0.015–3 ng/mL), and high cotinine concentrations (≥3 ng/mL) were used to categorize smoking.^[17]^ Alcohol intake was categorized into none and mild (fewer than 12 drinks annually), moderate (for women, up to 1 drink per day and for men, up to 2 drinks per day), and severe (drinking 2 or more drinks per day for women, 3 or more drinks per day for men, or 5 or more drinks a day).^[18]^ BMI categories were classified as under 25, between 25 and 30, and over 30 kg/m^2^. High blood pressure was defined as systolic readings over 140 mm Hg, diastolic readings above 90 mm Hg,^[19]^ taking medication for blood pressure, or self-reporting hypertension.

### 2.5. Statistical analysis

The categorical variables were presented as proportions and frequencies, and the Chi-square test was used to analyze them. Using weighted linear regression, mean values with standard errors were calculated for continuous variables. We performed trend tests on weight-adjusted waist index and DR to examine their linear relationship. Additionally, weight-adjusted waist index and DR were analyzed using a multivariate logistic regression analysis. Furthermore, age, sex, and BMI were examined as influences on weight-adjusted waist index levels and DR through subgroup analysis. In Model 1, all variables remained unchanged. In Model 2, adjustments were made for the primary demographic factors of age, gender, and ethnicity. Model 3 adjusted all covariates in the study. A smooth curve fitting technique was employed to examine the presence of nonlinearity. Data analysis was conducted using R version 4.2.2 (RStudio; https://www.r-project.org), EmpowerStats application (X&Y Solutions; http://www.empowerstats.com), and Stata 17.0 program (StataCorp, College Station). If the *P*-value is <.05, we consider the study to be statistically significant.

## 3. Results

### 3.1. Baseline characteristics

The research included 2001 individuals, averaging 59.4 years old (with a standard deviation of 13.8 years); among them, 1031 (50.6%) were men, and 970 (49.4%) were women. The average weight-adjusted waist index for all participants was 11.6 ± 0.8 cm/√kg. Table 1 displays the demographic traits of participants, categorized based on whether they have DR or not. As compared to the non-DR group, it was more likely that the DR group was non-Hispanic White and had a higher blood pressure, HbA1c levels, and weight-adjusted waist index, while having reduced albumin levels. Furthermore, participants with DR exhibited a lower poverty-to-income ratio, lower educational attainment, reduced alcohol consumption, and higher smoking rates.

**Table 1:** Overview of main traits of participants (n = 2001) categorized by presence or absence of diabetic retinopathy in the NHANES 2005–2017 study.

N	Total	No	Yes	*P* value
	2001	1624	377	
Age, yrs	59.4 ± 13.8	59.2 ± 13.9	60.5 ± 13.1	.1302
Sex, %				.7227
Male	50.6	50.7	49.7	
Female	49.4	49.3	50.3	
Race, %				.0042
Mexican American	9.4	9.7	7.8	
Other Hispanic	6.4	6.3	7.2	
Non-Hispanic White	60.8	62.2	54.2	
Non-Hispanic Black	14.7	14	17.9	
Other race – including multi-racial	8.7	7.9	12.9	
Education, %				<.001
<9th grade	10.4	9.6	14.2	
9–11th grade (Includes 12th grade with no diploma)	14	12.8	20.2	
High school graduate/GED or equivalent	24.5	23.9	27.3	
Some college or AA degree	30.5	32	22.9	
College graduate or above	19.6	20.5	15.3	
other	1	1.2	0.1	
Poverty income ratio, %				<.001
≤1.30	21.7	19.9	30.4	
1.31–3.50	39.1	38.6	41.7	
>3.50	31.4	33.6	20.4	
Missing	7.8	7.8	7.5	
Drink, %				<.001
None and mild	27.7	26.2	35.1	
Moderate	23	24.3	16.5	
Severe	30.8	32.1	24.4	
Not recorded	18.5	17.4	24.1	
HBP				.0023
Yes	72.2	71.1	77.4	
No	25.2	25.7	22.4	
Missing	2.6	3.1	0.2	
BMI, %				.5233
<25	11.2	11.4	10.6	
25–29.9	26.4	26.5	26.1	
≥30	62.2	62.1	63	
Not recorded	0.1	0.1	0.4	
SMOKCOT, %				.0494
<0.015 ng/mL	33.9	35	28.4	
0.015–3 ng/mL	44.2	43.1	49.8	
≥3 ng/mL	21	20.9	21.5	
Fasting glucose, mmol/L	8.8 ± 3.6	8.8 ± 3.4	9.3 ± 4.0	.0058
HbA1C, %	7.4 ± 1.7	7.3 ± 1.7	7.8 ± 1.9	<.001
Triglyceride, mmol/L	2.0 ± 2.3	1.9 ± 2.3	2.0 ± 2.4	.7489
Albumin, g/dL	4.1 ± 0.5	4.1 ± 0.5	4.0 ± 0.5	.0059
Total cholesterol, mmol/L	4.6 ± 1.2	4.6 ± 1.1	4.7 ± 1.3	.1864
LDL-cholesterol, mmol/L	2.6 ± 0.9	2.6 ± 0.9	2.6 ± 1.0	.4326
HDL-cholesterol, mmol/L	1.2 ± 0.4	1.2 ± 0.4	1.3 ± 0.4	.2236
Waist circumference, cm	110.6 ± 16.6	110.5 ± 16.7	111.0 ± 15.8	.5667
Weight, kg	91.7 ± 23.6	92.0 ± 23.7	90.6 ± 22.6	.3388
WWI	11.6 ± 0.8	11.6 ± 0.8	11.8 ± 0.8	.0008

Values are weighted mean ± standard deviation or weighted % (95 confidence interval). *P* values are weighted. Other races include American Indian or Alaska Native, Native Hawaiian or other Pacific Islander, and multiracial persons.

AA = associate of arts, BMI = body mass index, GED = General Educational Development, HbA1C = glycated hemoglobin, HBP = high blood pressure, HDL = high-density lipoprotein, LDL = low-density lipoprotein, NHANES = National Center and Examination Survey, WWI = weight-adjusted-waist index.

### 3.2. Relationship between the weight-adjusted waist index ratio and DR

**Figure 2.**
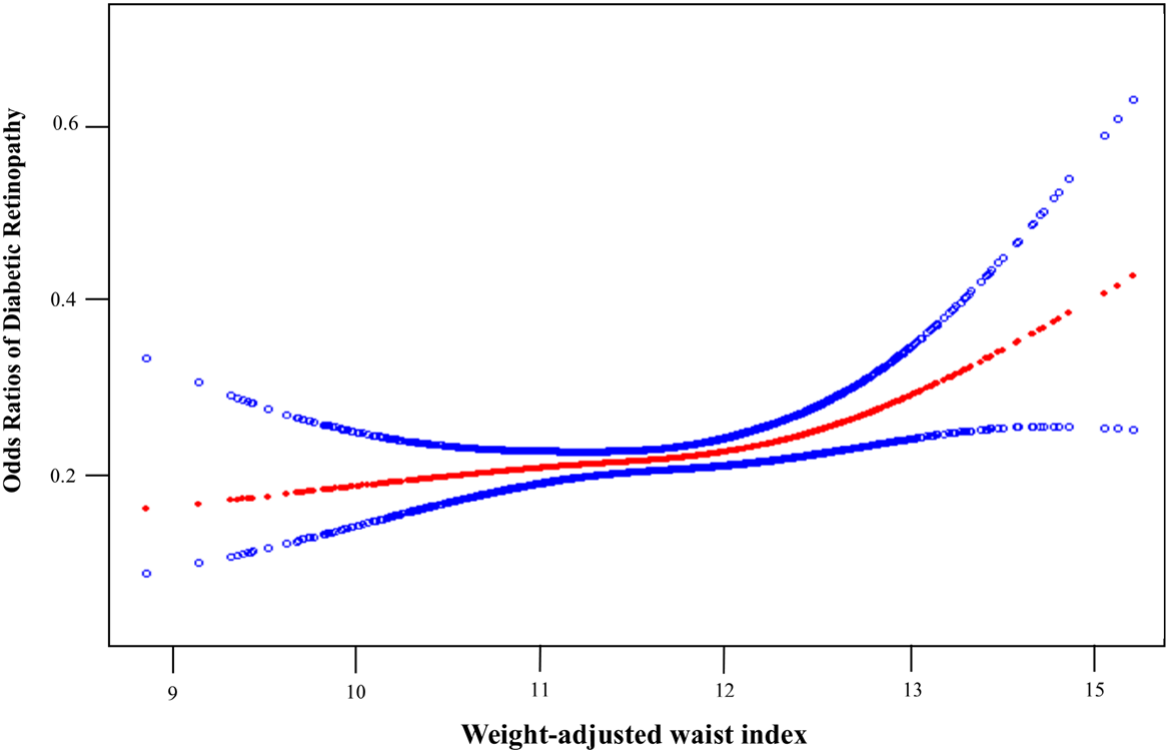
The association between weight-adjusted waist index and DR. The solid red line represents the smooth curve fit between variables. Blue bands represent the 95% confidence interval from the fit. DR = diabetic retinopathy

Analyses of logistic regression are presented in Table 2. After full adjustment, the previously noted positive association persisted as statistically significant (OR = 1.32, 95% CI: 1.02–.70), which means each unit increase in weight-adjusted waist index increased the risk of DR by 32%. It was evident that the favorable correlation continued even after the weight-adjusted waist index variable was divided into 4 groups in Model 2. The positive nonlinear relationship between weight-adjusted waist index and DR is further demonstrated in Figure 2, which shows an inflection point at 12.35. This finding is supported by the significant log-likelihood ratio presented in Table 3.

**Table 2:** Association between WWI and diabetic retinopathy (n = 2001) in the NHANES 2005–2017.

	Model 1 OR (95% CI) *P*-value	Model 2 OR (95% CI) *P*-value	Model 3 OR (95% CI) *P*-value
WWI (continuous)	1.31 (1.05–1.62) .014	1.39 (1.11–1.75) .004	1.32 (1.02–1.7) .034
WWI (quartile)			
Quartile 1	reference	reference	reference
Quartile 2	1.44 (0.92–2.26) .110	1.46 (0.91–2.34) .115	1.38 (0.83–2.30) .215
Quartile 3	1.35 (0.87–2.09) .186	1.42 (0.89–2.25) .138	1.43 (0.88–2.33) .153
Quartile 4	1.55 (0.99–2.43) .053	1.68 (1.05–2.07) .031	1.46 (0.86–2.48) .166
*P* trend	.085	.047	.187
Stratified by gender			
Men	1.14 (0.89–1.47) .290	1.25 (0.94–1.66) .125	1.29 (0.92–1.82) .143
Women	1.52 (1.09–2.11) .014	1.54 (1.1–2.15) .011	1.41 (1–1.99) .049
Stratified by age			
<40	1.34 (0.89–2.03) .159	1.47 (0.9–2.39) .125	3.83 (0.92–15.94) .065
40–49	0.95 (0.6–1.5) .827	1.12 (0.72–1.75) .616	0.72 (0.35–1.46)0.360
50–59	1.37 (0.83–2.28) .222	1.67 (1.03–2.71) .038	1.78 (1.07–2.95) .027
60–69	1.2 (0.74–1.94) .460	1.32 (0.79–2.21) .291	1.73 (1.06–2.84) .029
≥70	1.57 (1.07–2.3) .020	1.53 (1.04–2.26) .031	1.21 (0.81–1.79) .356
Stratified by race			
Mexican American	1.29 (0.8–2.07) .292	1.63 (1.01–2.62) .046	2.17 (1.2–3.92) .010
Other Hispanic	1.09 (0.56–2.13) .798	1.13 (0.58–2.21) .717	1.28 (0.6–2.71) .522
Non-Hispanic White	1.52 (1.09–2.11) .012	1.48 (1.06–2.07) .022	1.41 (0.96–2.08) .078
Non-Hispanic Black	1.33 (0.99–1.78) .060	1.38 (0.99–1.93) .06	1.09 (0.72–1.64) .686
Other race – including multi-racial	0.91 (0.48–1.71) .761	1.25 (0.68–2.31) .466	0.97 (0.44–2.18) .949

Model 1: non-adjusted model. Model 2: age, gender, and race were adjusted. Model 3: age, gender, race, educational level, poverty income ratio, alcohol consumption, high blood pressure, BMI, smoking level, fasting glucose, HbA1C, triglyceride, albumin, total cholesterol, LDL, and HDL were adjusted.

BMI = body mass index, HbA1C = glycated hemoglobin, HDL = high-density lipoprotein, LDL = low-density lipoprotein, NHANES = National Center and Examination Survey, WWI = weight-adjusted-waist index.

**Table 3:** Analysis of threshold effects of WWI on diabetic retinopathy.

Odds ratios of diabetic retinopathy	Adjusted OR (95% CI) *P*-value
Inflection point	12.35
WWI < 12.35	1.09 (0.86–1.38) .4627
WWI > 12.35	2.27 (1.28–4.03) .0050
Log likelihood ratio	0.039

Age, gender, race, educational level, poverty income ratio, alcohol consumption, high blood pressure, BMI, smoking level, fasting Glucose, HbA1C, triglyceride, albumin, totalcholesterol, LDL, HDL were adjusted.

BMI = body mass index, HbA1C = glycated hemoglobin, HDL = high-density lipoprotein, LDL = low-density lipoprotein, WWI = weight-adjusted-waist index.

### 3.3. Subgroup analysis

**Figure 3.**
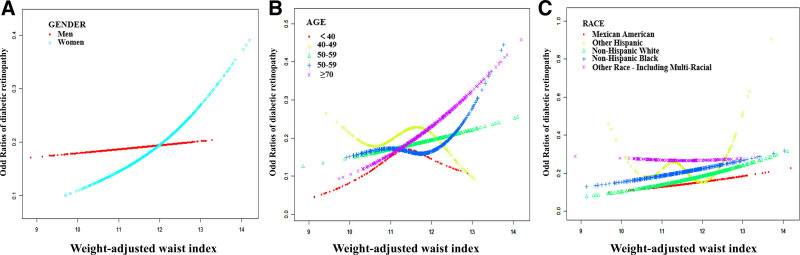
Relationship between weight-adjusted waist index and DR stratified by gender (A), age (B), and race (C). DR = diabetic retinopathy.

The link between weight-adjusted waist index and DR was evaluated using subgroup analyses. As demonstrated in the gender-specific subgroup analyses for Models 1 to 3, weight-adjusted waist index and DR are positively correlated for females (Table 2 and Fig. 3). Age-stratified subgroup analyses revealed that weight-adjusted waist index and DR showed a notable positive correlation for individuals aged 50 to 59 in both Model 2 and Model 3, for those aged 60 to 69 in Model 3, and for participants aged 70 and above in Models 1 to 2. The subgroup analysis categorized by race revealed a notable positive correlation between weight-adjusted waist index and DR among Non-Hispanic Whites in both Model 1 and Model 2, as shown in Table 2 and Figure 3.

## 4. Discussion

This research, involving 2001 individuals who met the lowest criteria, found a direct positive relationship between weight-adjusted waist index and DR. Furthermore, subgroup analysis revealed that this relationship was more pronounced among females; individuals aged 50 to 59, 60 to 69, and those aged 70 and above; as well as within the Non-Hispanic White demographic.

To our knowledge, this is the first cross-sectional human study investigating the weight-adjusted waist index and DR. Recent reports indicate that the weight-adjusted waist index is a novel metric for assessing obesity, which has been investigated across various disciplines, with a particular focus on its implications for cardiovascular health.^[11]^ We confirmed a notable link between the weight-adjusted waist index and DR. Furthermore, the linear positive relationship identified between the weight-adjusted waist index and DR persisted even after comprehensive adjustments in the fully controlled model. Subgroup analyses revealed a notably strong connection between the weight-adjusted waist index and DR, especially among women. This phenomenon may be attributable to the regulatory influence of estrogen on the differentiation and function of adipocytes,^[20]^ as well as the impact of estrogen on microvessels through mechanisms such as oxidative stress.^[21]^ Also, we found that the correlation between weight-adjusted waist index and DR varied by age and race, especially among older adults and non-Hispanic whites. We hypothesize that the process of aging could impact weight-adjusted waist index by altering lipid metabolism and the distribution of fat cells, with older age being acknowledged as a major risk factor for DR.^[22]^ Non-Hispanic Blacks and Mexican Americans with type 2 diabetes have higher rates and severity of DR compared to non-Hispanic Whites, according to a population survey conducted from 1994 to 1998.^[23]^ However, a cross-sectional survey from 2011 to 2018 showed a rising trend in obesity and body fat measures among Non-Hispanic Whites,^[24]^ which might partly clarify the findings of the current study.

Numerous factors contribute to the risk of developing DR, including well-established determinants such as elevated blood pressure and inadequate glycemic control.^[25]^ Growing research indicates that being overweight is a major contributor to diabetes-related complications such as DR.^[26]^ Numerous indicators have been employed to assess obesity, including BMI and WC, which are traditionally regarded as standard measures of obesity. A prospective cohort study with 30,541 UK participants indicated that higher BMI and WC could be potential causal risk factors for diabetic microvascular issues, with each unit rise in BMI and WC linked to a 9% and 11% increase, respectively, in the likelihood of developing DR.^[27]^ In a study using two-sample Mendelian randomization, overall obesity, estimated by BMI, and central obesity, assessed by waist and hip measurements, increased the likelihood of developing diabetes.^[28]^ Although there is growing proof connecting conventional obesity markers with DR, the obesity paradox remains.^[29,30]^ In the debate, conventional metrics do not distinguish between fat mass and muscle mass, which is a key factor.^[31,32]^ An alternative explanation is that the correlations between different body measurements make it difficult to pinpoint biologically determined disease risks, thus hindering precise disease forecasting.^[10]^ In addition to WC’s benefits, weight-adjusted waist index reduces its correlation with BMI, allowing it to accurately detect obesity regardless of total body weight,^[33]^ which is considered to be one of the major risk factors for type 2 diabetes.^[34]^ Additionally, the weight-adjusted waist index could be a better indicator of the metabolic syndrome and high blood pressure that are associated with DR.^[35,36]^ While various indicators for evaluating obesity exist, it is noteworthy that certain novel anthropometric indices, such as the Visceral Adiposity Index, are derived from relatively complex mathematical formulas, which can be inconvenient for practical application, whereas the weight-adjusted waist index circumvents this issue.^[37]^ The weight-adjusted waist index is also relevant to diverse racial and demographic groups and may exhibit greater stability and reliability in cross-racial or multicenter studies.^[38]^ In recent studies, weight-adjusted waist index outperformed both BMI and WC as a significant predictor of a wide range of diseases.^[37,39]^ To sum up, weight-adjusted waist index shows excellent results in forecasting disease risk, making it an important tool for healthcare providers to consider.

Several potential mechanisms could explain the observed positive association between weight-adjusted waist index and DR. Firstly, an elevated weight-adjusted waist index may indicate dysfunction in adipose tissue, which in turn could facilitate the release of various proinflammatory cytokines and enhance oxidative stress in individuals.^[40]^ These 2 mechanisms have been demonstrated to constitute the primary pathogenesis of DR.^[41]^ For instance, triglyceride-rich lipoproteins can induce a direct pro-inflammatory response by activating blood monocytes, which may result in retinal microvascular injury.^[42]^ Furthermore, in people with obesity, fat tissue emits higher amounts of reactive oxygen species, potentially harming the area around retinal blood vessels and leading to the onset of DR.^[43]^ Secondly, being overweight has been recognized as a factor leading to insulin resistance, a condition closely linked to the development of DR.^[44]^ Obesity causes the enlargement and malfunction of fat tissues, resulting in persistent low-level systemic inflammation driven by pro-inflammatory cytokines from the expanded fat tissue, which hinders insulin function in the insulin signaling pathway and disturbs glucose balance.^[45]^ These metabolic disruptions have been linked to the thinning of the retinal outer nuclear layer’s optical layers and the photoreceptor myoid zone, indicating initial metabolic changes in the retina related to diabetes.^[46]^ Third, comorbid conditions associated with obesity, including hypertriglyceridemia, impaired glucose tolerance, and hypertension, further elevate the risk of developing DR.^[36,47]^

There are several benefits to this study, especially the fact that the data are drawn from a survey database that accurately portrays the U.S. population, making the study more credible and relevant. Throughout the research, we accounted for all variables and conducted additional subgroup analyses based on gender, age, and race to explore the connection between weight-adjusted waist index and DR. Nevertheless, the study exhibits certain limitations. The causal relationship between weight-adjusted waist index and DR could not be ascertained due to the inherent limitations of the cross-sectional study design. The enhancement of diagnostic criteria for DR persists, exemplified by the practice of capturing retinal images of both eyes. As well, the weight-adjusted waist index, the new obesity measure, has not been widely accepted as a method of measuring obesity and central obesity in clinical settings, in contrast to BMI and WC. Additional studies are needed to clarify the advantages and disadvantages of weight-adjusted waist index across different medical scenarios. In conclusion, our study leverages the NHANES database, which pertains to the U.S. population, thereby limiting its geographical applicability. Further empirical research is needed to determine if there is a connection between weight-adjusted waist index and DR in various national or regional groups.

## 5. Conclusions

The current study indicates a positive correlation between weight-adjusted waist index and DR. It is possible that our findings could help prevent and treat DR in the future. However, more research is required to confirm our findings.

## Author contributions

**Conceptualization:** Junxian Lei, Jun Peng, Qinghua Peng.

**Data curation:** Junxian Lei, Kun Lian, Pai Zhou.

**Formal analysis:** Junxian Lei, Kun Lian, Pai Zhou.

**Funding acquisition:** Jun Peng, Qinghua Peng.

**Methodology:** Junxian Lei, Kun Lian, Pai Zhou, Qinghua Peng.

**Project administration:** Jun Peng, Qinghua Peng.

**Software:** Junxian Lei, Kun Lian, Pai Zhou.

**Supervision:** Jun Peng, Qinghua Peng.

**Validation:** Qinghua Peng.

**Visualization:** Kun Lian, Pai Zhou.

**Writing – original draft:** Junxian Lei.

**Writing – review & editing:** Junxian Lei, Kun Lian, Pai Zhou, Jun Peng, Qinghua Peng.

## References

[1] Nawaz IM Rezzola S Cancarini A. Human vitreous in proliferative diabetic retinopathy: characterization and translational implications. Prog Retin Eye Res. 2019;72:100756. 30951889 10.1016/j.preteyeres.2019.03.002

[2] Purola PKM Ojamo MUI Gissler M Uusitalo HMT. Changes in visual impairment due to diabetic retinopathy during 1980-2019 based on nationwide register data. Diabetes Care. 2022;45:2020–7. 35838317 10.2337/dc21-2369 PMC9472510

[3] Flaxman SR Bourne RRA Resnikoff S. Global causes of blindness and distance vision impairment 1990-2020: a systematic review and meta-analysis. Lancet Glob Health. 2017;5:e1221–34. 29032195 10.1016/S2214-109X(17)30393-5

[4] De Lorenzo A Gratteri S Gualtieri P Cammarano A Bertucci P Di Renzo L. Why primary obesity is a disease. J Transl Med. 2019;17:169. 31118060 10.1186/s12967-019-1919-y PMC6530037

[5] Javed A Jumean M Murad MH. Diagnostic performance of body mass index to identify obesity as defined by body adiposity in children and adolescents: a systematic review and meta-analysis. Pediatr Obes. 2015;10:234–44. 24961794 10.1111/ijpo.242

[6] Ness-Abramof R Apovian CM. Waist circumference measurement in clinical practice. Nutr Clin Pract. 2008;23:397–404. 18682591 10.1177/0884533608321700

[7] Mahmoud I Al-Wandi AS Gharaibeh SS Mohamed SA. Concordances and correlations between anthropometric indices of obesity: a systematic review. Public Health. 2021;198:301–6. 34507136 10.1016/j.puhe.2021.07.042

[8] Thomas EL Frost G Taylor-Robinson SD Bell JD. Excess body fat in obese and normal-weight subjects. Nutr Res Rev. 2012;25:150–61. 22625426 10.1017/S0954422412000054

[9] Park Y Kim NH Kwon TY Kim SG. A novel adiposity index as an integrated predictor of cardiometabolic disease morbidity and mortality. Sci Rep. 2018;8:16753. 30425288 10.1038/s41598-018-35073-4 PMC6233180

[10] Qin Z Chang K Yang Q Yu Q Liao R Su B. The association between weight-adjusted-waist index and increased urinary albumin excretion in adults: a population-based study. Front Nutr. 2022;9:941926. 36034904 10.3389/fnut.2022.941926 PMC9412203

[11] Ding C Shi Y Li J. Association of weight-adjusted-waist index with all-cause and cardiovascular mortality in China: a prospective cohort study. Nutr Metab Cardiovasc Dis. 2022;32:1210–7. 35277327 10.1016/j.numecd.2022.01.033

[12] Liu Y Liu X Zhang S. Association of anthropometric indices with the development of diabetes among hypertensive patients in China: a cohort study. Front Endocrinol (Lausanne). 2021;12:736077. 34675879 10.3389/fendo.2021.736077 PMC8525507

[13] Wan H Wang Y Xiang Q. Associations between abdominal obesity indices and diabetic complications: Chinese Visceral Adiposity Index and neck circumference. Cardiovasc Diabetol. 2020;19:118. 32736628 10.1186/s12933-020-01095-4 PMC7395356

[14] Simó R Hernández C. What else can we do to prevent diabetic retinopathy. Diabetologia. 2023;66:1614–21. 37277664 10.1007/s00125-023-05940-5 PMC10390367

[15] Wang H Guo Z Xu Y. Association of monocyte-lymphocyte ratio and proliferative diabetic retinopathy in the U.S. population with type 2 diabetes. J Transl Med. 2022;20:219. 35562757 10.1186/s12967-022-03425-4 PMC9102352

[16] Sun XJ Zhang GH Guo CM. Associations between psycho-behavioral risk factors and diabetic retinopathy: NHANES (2005-2018). Front Public Health. 2022;10:966714. 36187629 10.3389/fpubh.2022.966714 PMC9521717

[17] Hou W Chen S Zhu C Gu Y Zhu L Zhou Z. Associations between smoke exposure and osteoporosis or osteopenia in a US NHANES population of elderly individuals. Front Endocrinol (Lausanne). 2023;14:1074574. 36817605 10.3389/fendo.2023.1074574 PMC9935577

[18] Xie ZQ Li HX Wang BK. Trends in prevalence and all-cause mortality of metabolic dysfunction-associated fatty liver disease among adults in the past three decades: results from the NHANES study. Eur J Intern Med. 2023;110:62–70. 36754655 10.1016/j.ejim.2023.01.029

[19] Fryar CD Ostchega Y Hales CM Zhang G Kruszon-Moran D. Hypertension prevalence and control among adults: United States, 2015-2016. NCHS Data Brief. 2017;289:1–8. 29155682

[20] de Medeiros SF Rodgers RJ Norman RJ. Adipocyte and steroidogenic cell cross-talk in polycystic ovary syndrome. Hum Reprod Update. 2021;27:771–96. 33764457 10.1093/humupd/dmab004

[21] Maric-Bilkan C. Sex differences in micro- and macro-vascular complications of diabetes mellitus. Clin Sci (Lond). 2017;131:833–46. 28424377 10.1042/CS20160998

[22] Nguyen TT Corvera S. Adipose tissue as a linchpin of organismal ageing. Nat Metab. 2024;6:793–807. 38783156 10.1038/s42255-024-01046-3 PMC11238912

[23] Harris MI Klein R Cowie CC Rowland M Byrd-Holt DD. Is the risk of diabetic retinopathy greater in non-Hispanic blacks and Mexican Americans than in non-Hispanic whites with type 2 diabetes? A U.S. population study. Diabetes Care. 1998;21:1230–5. 9702425 10.2337/diacare.21.8.1230

[24] Liu B Du Y Wu Y Snetselaar LG Wallace RB Bao W. Trends in obesity and adiposity measures by race or ethnicity among adults in the United States 2011-18: population based study. BMJ. 2021;372:n365. 33727242 10.1136/bmj.n365 PMC7961695

[25] Pan X He H Bao Y. Chinese expert consensus on the management of hypertension in adults with type 2 diabetes. J Evid Based Med. 2024;17:851–64. 39529557 10.1111/jebm.12655

[26] Rohm TV Meier DT Olefsky JM Donath MY. Inflammation in obesity, diabetes, and related disorders. Immunity. 2022;55:31–55. 35021057 10.1016/j.immuni.2021.12.013 PMC8773457

[27] Huang Y Zhang X Li B. Association of BMI and waist circumference with diabetic microvascular complications: a prospective cohort study from the UK Biobank and Mendelian randomization analysis. Diabetes Res Clin Pract. 2023;205:110975. 37884062 10.1016/j.diabres.2023.110975

[28] Zheng C Wei X Cao X. The causal effect of obesity on diabetic retinopathy: a two-sample Mendelian randomization study. Front Endocrinol (Lausanne). 2023;14:1108731. 37077358 10.3389/fendo.2023.1108731 PMC10106681

[29] Levitsky LL Drews KL Haymond M. The obesity paradox: retinopathy, obesity, and circulating risk markers in youth with type 2 diabetes in the TODAY Study. J Diabetes Complications. 2022;36:108259. 36150365 10.1016/j.jdiacomp.2022.108259 PMC12396272

[30] Chen J Li YT Niu Z. Investigating the causal association of generalized and abdominal obesity with microvascular complications in patients with type 2 diabetes: a community-based prospective study. Diabetes Obes Metab. 2024;26:2796–810. 38695216 10.1111/dom.15598

[31] Hainer V Aldhoon-Hainerová I. Obesity paradox does exist. Diabetes Care. 2013;36(Suppl 2):S276–81. 23882059 10.2337/dcS13-2023 PMC3920805

[32] Antonopoulos AS Oikonomou EK Antoniades C Tousoulis D. From the BMI paradox to the obesity paradox: the obesity-mortality association in coronary heart disease. Obes Rev. 2016;17:989–1000. 27405510 10.1111/obr.12440

[33] Ye J Hu Y Chen X. Association between the weight-adjusted waist index and stroke: a cross-sectional study. BMC Public Health. 2023;23:1689. 37658310 10.1186/s12889-023-16621-8 PMC10472709

[34] Lee YJ Kim JJ Kim J Cho DW Won JY. The correlation between waist circumference and the pro-inflammatory adipokines in diabetic retinopathy of type 2 diabetes patients. Int J Mol Sci. 2023;24:2036. 36768360 10.3390/ijms24032036 PMC9917192

[35] Mbata O Abo El-Magd NF El-Remessy AB. Obesity, metabolic syndrome and diabetic retinopathy: beyond hyperglycemia. World J Diabetes. 2017;8:317–29. 28751954 10.4239/wjd.v8.i7.317 PMC5507828

[36] Lopes de Faria JB Silva KC Lopes de Faria JM. The contribution of hypertension to diabetic nephropathy and retinopathy: the role of inflammation and oxidative stress. Hypertens Res. 2011;34:413–22. 21228783 10.1038/hr.2010.263

[37] Cao S Hu X Shao Y. Relationship between weight-adjusted-waist index and erectile dysfunction in the United State: results from NHANES 2001-2004. Front Endocrinol (Lausanne). 2023;14:1128076. 37181040 10.3389/fendo.2023.1128076 PMC10167952

[38] Kim JY Choi J Vella CA Criqui MH Allison MA Kim NH. Associations between weight-adjusted waist index and abdominal fat and muscle mass: multi-ethnic study of atherosclerosis. Diabetes Metab J. 2022;46:747–55. 35350091 10.4093/dmj.2021.0294 PMC9532169

[39] Xie F Xiao Y Li X Wu Y. Association between the weight-adjusted-waist index and abdominal aortic calcification in United States adults: results from the National Health and Nutrition Examination Survey 2013-2014. Front Cardiovasc Med. 2022;9:948194. 36186965 10.3389/fcvm.2022.948194 PMC9515490

[40] Karam BS Chavez-Moreno A Koh W Akar JG Akar FG. Oxidative stress and inflammation as central mediators of atrial fibrillation in obesity and diabetes. Cardiovasc Diabetol. 2017;16:120. 28962617 10.1186/s12933-017-0604-9 PMC5622555

[41] Chen C Ding P Yan W. Pharmacological roles of lncRNAs in diabetic retinopathy with a focus on oxidative stress and inflammation. Biochem Pharmacol. 2023;214:115643. 37315816 10.1016/j.bcp.2023.115643

[42] Forrester JV Kuffova L Delibegovic M. The role of inflammation in diabetic retinopathy. Front Immunol. 2020;11:583687. 33240272 10.3389/fimmu.2020.583687 PMC7677305

[43] Kang Q Yang C. Oxidative stress and diabetic retinopathy: molecular mechanisms, pathogenetic role and therapeutic implications. Redox Biol. 2020;37:101799. 33248932 10.1016/j.redox.2020.101799 PMC7767789

[44] Katsiki N Anagnostis P Kotsa K Goulis DG Mikhailidis DP. Obesity, metabolic syndrome and the risk of microvascular complications in patients with diabetes mellitus. Curr Pharm Des. 2019;25:2051–9. 31298151 10.2174/1381612825666190708192134

[45] Ahmed B Sultana R Greene MW. Adipose tissue and insulin resistance in obese. Biomed Pharmacother. 2021;137:111315. 33561645 10.1016/j.biopha.2021.111315

[46] Rauscher FG Elze T Francke M. Glucose tolerance and insulin resistance/sensitivity associate with retinal layer characteristics: the LIFE-Adult-Study. Diabetologia. 2024;67:928–39. 38431705 10.1007/s00125-024-06093-9 PMC10954961

[47] Wat N Wong RL Wong IY. Associations between diabetic retinopathy and systemic risk factors. Hong Kong Med J. 2016;22:589–99. 27779095 10.12809/hkmj164869

